# Artificial intelligence-driven phage therapy in veterinary medicine: an adaptive One Health strategy to mitigate antimicrobial resistance in livestock systems

**DOI:** 10.3389/fvets.2026.1829777

**Published:** 2026-06-24

**Authors:** Vincenzo Cuteri, Chiara Storoni, Shengliang Cao, Yubao Li

**Affiliations:** 1 School of Biosciences and Veterinary Medicine, University of Camerino, Matelica, Italy; 2 School of Pharmaceutical Sciences and Food Engineering, Liaocheng University, Liaocheng, China

**Keywords:** antimicrobial resistance, artificial intelligence, bacteriophage therapy, evolutionary modeling, livestock production, One Health, phage–host interaction, precision livestock farming

## Abstract

Antimicrobial resistance (AMR) in animal production systems is a major structural driver of the global resistance crisis. Food-producing animals account for the majority of global antimicrobial consumption, generating sustained selective pressure across livestock, environmental, and zoonotic bacterial reservoirs. Intensive poultry, swine, cattle, and aquaculture systems amplify pathogen transmission and accelerate resistance emergence. Bacteriophage therapy offers a species-specific, microbiome-preserving alternative to conventional antibiotics; however, large-scale veterinary implementation has historically been constrained by challenges including strain-level host prediction, resistance evolution, biosafety considerations, manufacturing scalability, economic feasibility, and regulatory adaptation. Recent advances in artificial intelligence (AI) show promise for enabling precision veterinary phage therapy, though most applications remain at the computational proof-of-concept or preclinical stage. Deep learning and graph-based genomic models have demonstrated high accuracy on benchmark datasets, reinforcement learning has been explored in computational models for cocktail optimization, and AI-assisted genomic screening can enhance biosafety assessment. Integration with real-time AMR surveillance could potentially facilitate adaptive deployment strategies, subject to field validation. Economic modeling suggests that moderate reductions in metaphylactic antibiotic use could yield production and public health benefits, though these estimates remain illustrative. This review synthesizes current evidence on AI-guided phage discovery, epidemiological modeling, microbiome modulation, horizontal gene transfer risk assessment, economic evaluation, and regulatory innovation. Within a One Health framework, adaptive AI-guided phage platforms represent a high-leverage strategy for reducing antimicrobial dependence, provided that critical knowledge gaps are addressed.

## Graphical abstract

**Figure:**
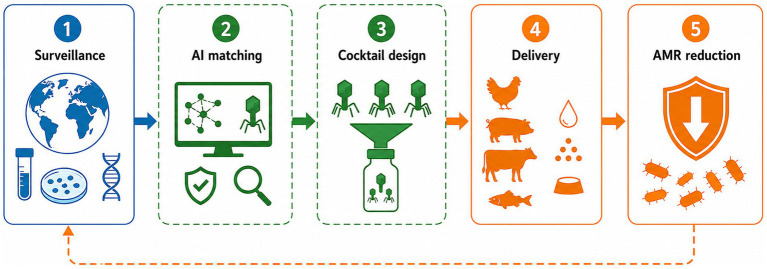
Simplified workflow of Al-guided phage therapy in livestock: (1) pathogen surveillance (sampling, genomics), (2) Al-mediated host matching and biosafety screening, (3) evolution-aware cocktail design, (4) delivery (feed/water), and (5) AMR reduction. Feedback loop indicates adaptive updating based on outcome monitoring. Dashed boxes denote steps that remain at computational proof-of-concept or speculative stages pending field validation.

## 1 Quantitative burden of antimicrobial use and the central role of veterinary medicine

### 1.1 Quantitative burden of antimicrobial use

Antimicrobial resistance in animal production systems is quantitatively substantial and structurally embedded within modern livestock agriculture. Globally, food-producing animals account for approximately 73% of antimicrobial consumption (1). Modeling estimates indicate that antimicrobial use in livestock exceeded 93,000 tons in 2017 and may continue increasing in middle-income countries (2). Critically important antimicrobials for human medicine—including macrolides, fluoroquinolones, and third-generation cephalosporins—remain widely used in several livestock sectors, generating cross-resistance risks at the human-animal interface (3). Resistant non-typhoidal *Salmonella*, *Campylobacter jejuni*, extended-spectrum β-lactamase (ESBL)-producing *Escherichia coli*, and livestock-associated methicillin-resistant *Staphylococcus aureus* (LA-MRSA) have all been linked to antimicrobial use in food animals and subsequent zoonotic transmission (3, 4). The 2019 Global Burden of Bacterial AMR (66) study estimated 4.95 million deaths associated with bacterial resistance (4). Ecological and genomic evidence demonstrates bidirectional transmission of resistance determinants across animal, environmental, and human reservoirs (5, 6).

### 1.2 Veterinary medicine at the core of the AMR crisis

While AMR is often framed as a hospital-centered issue, veterinary production systems play a structurally central role (4, 7). In poultry, swine, and aquaculture industries, antimicrobials are widely used for therapeutic treatment, metaphylaxis, and prophylaxis. Resistant pathogens emerging in these systems—including *Salmonella enterica*, *Campylobacter jejuni*, avian pathogenic *E. coli* (APEC), and LA-MRSA—represent direct threats to food safety, animal welfare, and public health (3).

Recent surveillance data from low- and middle-income countries show alarming trends. In Moroccan poultry, Oubouyahia et al. (8) reported high levels of resistance to critically important antimicrobials. Sadi et al. (9) documented ESBL-producing *E. coli* in Algerian cattle. Shehata et al. (10) reported that *Klebsiella pneumoniae* from bovine mastitis exhibited high resistance to multiple antibiotic classes. In goats, Javed et al. (11) characterized β-lactam-resistant *S. aureus* from mastitis. In broilers, Aslantaş and Türkyılmaz (12) identified integrons in *Salmonella Infantis* clinical isolates. A recent meta-analysis found that for major livestock pathogens, clinical effectiveness declined from approximately 85% in 2000 to 65% in 2018—meaning that approximately one in three courses of antibiotic treatment may now fail due to resistance (5, 13). This figure varies substantially by region and pathogen-drug combination.

### 1.3 Economic consequences of AMR in livestock

Resistant bacterial outbreaks are associated with increased mortality, reduced feed conversion efficiency, prolonged production cycles, and trade restrictions. The FAO (14) estimates that AMR could reduce global livestock productivity by 2–5% annually by 2050. In broiler poultry, resistant colibacillosis outbreaks have been associated with 5–15% increased mortality; in swine, resistant infections can increase treatment costs by 200–400%; in dairy cattle, resistant mastitis is associated with 10–20% reduction in milk yield (3, 13).

### 1.4 Bacteriophage therapy as an alternative approach

Bacteriophage therapy offers species-specific pathogen targeting while preserving microbiome integrity (15, 16). However, deployment in livestock faces biological, computational, regulatory, and economic constraints. Artificial intelligence (AI) now provides computational tools that have shown potential to address some of these constraints, though most applications remain at the computational proof-of-concept stage.

The novel contribution of this review is threefold: (1) a systematic synthesis of AI applications specific to veterinary phage therapy with explicit classification of validation status; (2) a critical evaluation of barriers to implementation in livestock systems; and (3) a research roadmap grounded in current capabilities rather than aspirational projections. A major limitation is that most AI tools for phage-host prediction have been developed and validated using human clinical isolates. Livestock-specific validation is extremely limited, with no published field trials of AI-guided phage therapy in commercial livestock systems as of March 2026.

## 2 AI-guided phage discovery and host prediction

### 2.1 Metagenomic mining of agricultural phage reservoirs

Agricultural ecosystems contain vast viral diversity, much of which remains uncharacterized (“viral dark matter”) (17). Alignment-free models such as k-mer frequency classifiers and deep learning approaches have improved phage identification (18, 19). Convolutional neural networks (CNNs) extract local motif features, while recurrent neural networks (RNNs) capture long-range dependencies. Transformer-based genomic models (e.g., DNABERT) capture higher-order sequence dependencies (20). **[Computational proof-of-concept]**—these models have been demonstrated on benchmark datasets, but performance on livestock-derived samples requires validation.

In agricultural contexts, these architectures have been proposed to enable: (i) rapid identification of strictly lytic phages; (ii) detection of virulence or antimicrobial resistance genes; (iii) prediction of replication strategy; and (iv) prioritization of candidates for wet-lab validation (19, 21). Integration of CRISPR spacer databases further enhances host linkage prediction (18).

### 2.2 Strain-level host prediction

Strain-level specificity is essential in livestock medicine. Recent computational frameworks predict phage-host interactions directly from genomic features. Machine learning models have achieved high predictive accuracy on benchmark datasets (22, 23). Graph neural networks (GNNs) enable interaction prediction using relational modeling (24). Structural modeling via AlphaFold has enabled *in silico* analysis of receptor-binding protein docking (25). Gaborieau et al. (22) demonstrated AUROC values of 0.92–0.95 on human clinical isolate datasets. However, no study has validated these models on livestock-associated pathogens under field conditions *[preclinical evidence]*.

Mechanistically informed models incorporate receptor-binding protein sequence motifs, adsorption factor genes, bacterial surface polysaccharide biosynthesis clusters, and prophage defense systems. Graph-based models represent phages and bacterial strains as nodes within interaction networks, enabling prediction of unobserved edges through link prediction algorithms (24).

### 2.3 Evolution-aware AI frameworks

#### 2.3.1 Reinforcement learning for cocktail optimization

Phage cocktail design can be formalized as a multi-objective optimization problem: maximize strain coverage, minimize resistance emergence, minimize cross-resistance, preserve microbiome stability, and ensure manufacturability. Reinforcement learning (RL) frameworks have been explored in computational proof-of-concept studies (26, 27). The AI agent evaluates treatment strategies within simulated population models and updates policies to minimize resistance trajectories. This has been demonstrated in computational simulations (26) but not validated in live poultry **[computational proof-of-concept]**.

#### 2.3.2 Coevolutionary modeling

Bacteria-phage interactions follow either arms race dynamics or fluctuating selection dynamics (26, 28). Machine learning models trained on longitudinal surveillance data could potentially detect early signals of resistance allele expansion, but this remains **[speculative]**.

### 2.4 Evaluation standards and validation gaps

Reported performance metrics for AI phage prediction tools should be interpreted with caution. DeepHost (19) reports AUROC of 0.92–0.95 on benchmark datasets, but these datasets are predominantly human clinical isolates. No published study has externally validated any AI phage-host prediction model on an independent livestock-derived dataset. Public databases contain <15% sequences from livestock-associated samples, with overrepresentation of North American and European isolates. Most current models function as “black boxes” without biological interpretability. Recommended validation standards include leave-one-farm-out cross-validation and prospective validation on geographically distinct isolates.

### 2.5 Geographic and economic bias in training data

Beyond the general underrepresentation of livestock-associated sequences, significant geographic bias exists. Public databases contain >80% of sequences from North America and Europe, whereas livestock production is expanding most rapidly in Asia, Africa, and South America. This geographic mismatch has direct implications for AI model generalizability: genomic features of bacterial strains (e.g., *E. coli* ST131, *Salmonella* serovars) vary regionally due to differences in antimicrobial use patterns, farming practices, and climate. An AI model trained predominantly on European isolates may perform poorly on Asian isolates of the same species. Furthermore, low- and middle-income countries (LMICs) bear the highest AMR burden yet have the fewest genomic surveillance resources, creating a feedback loop where the populations most in need of AI-guided phage therapy are least represented in training data. Addressing this gap requires targeted sequencing efforts in LMICs and open-access data sharing agreements.

Table 1 summarizes the validation status of AI approaches for veterinary phage therapy.

**Table 1:** Validation status of artificial intelligence approaches for veterinary phage therapy.

AI approach	Validation status	Livestock-specific studies (*n*)	Key gaps
Phage identification from metagenomes (CNNs, RNNs, Transformers)	Computational proof-of-concept	0	No validation on livestock-derived metagenomic samples; benchmark datasets from human gut/environment
Strain-level phage-host prediction (GNNs, random forests)	Preclinical evidence (human clinical isolates)	0	Validated on human pathogens; livestock pathogen validation absent; geographic bias; no validation on LMIC isolates
AlphaFold-based RBP-receptor docking	Computational proof-of-concept	0	Requires structural data for livestock bacterial receptors; not validated for veterinary phages
Reinforcement learning for cocktail optimization	Computational proof-of-concept	0	Simulated only; no *in vivo* or field validation; resistance evolution parameters unknown
Coevolutionary modeling	Speculative	0	No livestock-specific longitudinal data for training
AI-assisted biosafety screening	Computational proof-of-concept	0	Accuracy on livestock phages unknown; no standardized genome safety criteria
Digital twin / precision livestock integration	Speculative	0	No implementation in any livestock species

Validation status definitions: **Confirmed** = validated in livestock under field conditions; **Preclinical evidence** = validated in vitro, ex vivo, or in small animal models; **Computational proof-of-concept** = demonstrated on benchmark datasets only; **Speculative** = conceptual framework without published implementation.

## 3 Epidemiological modeling in high-density livestock systems

Livestock production systems differ fundamentally from human clinical settings because interventions are applied at the population level. In intensive poultry, swine, and aquaculture systems, bacterial transmission dynamics are characterized by high contact rates, short generation times, and substantial environmental reservoirs (26, 27). **[Illustrative models]**—the mathematical models presented are illustrative frameworks adapted from foundational phage ecology literature (27). No phage-bacteria coevolution model has been prospectively validated in commercial livestock systems.

### 3.1 Population-level transmission dynamics

Population-level models describe bacterial-phage interactions (27):


Bacterial dynamics:dB/dt=rB(1−B/K)−φPB−μB

Phage dynamics:dP/dt=βφPB−ωP

Where *B* = bacterial density, *P* = phage density, *r* = bacterial growth rate, *K* = carrying capacity, *φ* = adsorption rate, *β* = burst size, *ω* = phage decay rate.

Key assumptions (violated in real farms): well-mixed population, constant adsorption rate, instantaneous lysis, no spatial structure. In livestock systems, these models must be extended to incorporate an environmental compartment:

d*E*/d*t* = *αB* − *δE*.

Where *E* = environmental bacterial density, *α* = shedding rate, *δ* = environmental decay rate.

**Example (poultry litter):** *Salmonella* can persist in litter for weeks. Bacteria shed in feces contaminate litter, where they can survive and replicate. This environmental reservoir means that even if all birds are treated successfully, recontamination from litter can occur.

### 3.2 Modeling resistance emergence

Phage resistance arises through receptor modification, CRISPR spacer acquisition, restriction-modification systems, or abortive infection pathways (15, 26). Partitioning into susceptible (*Bs*) and resistant (*Br*) subpopulations:


dBs/dt=rBs(1−(Bs+Br)/K)−φPBs−μBs

dBr/dt=r(1−c)Br(1–(Bs+Br)/K)+mBs

Where *c* = fitness cost of resistance, *m* = mutation rate. Parameter ranges are drawn from *in vitro* studies (29) and theoretical literature. No published study has empirically estimated these parameters for phage resistance in commercial livestock settings.

### 3.3 Reinforcement learning for adaptive cocktail deployment

**[Conceptual framework—speculative]**—the objective is to minimize cumulative bacterial load and resistance frequency over time. In RL, the system state includes bacterial strain distribution, resistance allele frequency, and environmental contamination. The action space includes phage selection, dosing frequency, and delivery route. No RL-driven adaptive phage deployment has been implemented in livestock.

### 3.4 Integration with precision livestock farming

Modern production facilities incorporate sensor-based monitoring systems measuring temperature, humidity, ammonia, water intake, and feed conversion ratio (30, 31). Digital twin models for phage-bacteria dynamics remain *[speculative]*; no published implementation exists in any livestock species.

### 3.5 Validation status of epidemiological models

No published study has validated any phage-bacteria coevolution model in commercial livestock against field outcomes. The general principle that resistance emerges during phage therapy is confirmed from in vitro and small-animal studies. However, specific predictions (time-to-resistance, required dosing frequency) remain unvalidated.

## 4 Microbiome and resistome modulation

### 4.1 Microbiome stability in intensive production systems

The gastrointestinal microbiome plays essential roles in nutrient metabolism, feed conversion, colonization resistance, and immune maturation. Metaphylactic antibiotic administration reduces microbial diversity and promotes expansion of antimicrobial resistance genes (3, 32, 33). Preclinical studies demonstrate that phage therapy can reduce pathogenic bacterial loads without significantly altering overall microbiome diversity indices compared to antibiotic-treated controls (34, 35). In murine and avian models, phage administration selectively reduced pathogen abundance while preserving anaerobic commensals **[preclinical evidence]**. Livestock-specific validation is lacking.

Dysbiosis is associated with impaired feed conversion ratios. In broilers, a 10% reduction in gut microbiome diversity has been associated with a 3–5% increase in feed conversion ratio (36). Similar relationships exist in pigs (37) and feedlot cattle (38).

### 4.2 Resistome dynamics under phage versus antibiotic pressure

The livestock gut resistome is a major reservoir of antimicrobial resistance genes. Antibiotic exposure increases ARG abundance even when pathogen suppression is achieved (5, 32). Phage therapy alters selective pressure differently: it targets specific hosts, does not directly select for multidrug resistance, and resistance often involves receptor modification rather than ARG acquisition (29). Longitudinal metagenomic monitoring can quantify resistome shifts, but livestock-specific data are limited.

### 4.3 Environmental microbiome and slurry ecosystems

Livestock-associated microbiomes extend to manure lagoons, bedding materials, and aquaculture water columns. These environments serve as reservoirs of both pathogens and ARGs (6). Phage application may reduce environmental pathogen load without introducing chemical residues. AI-driven ecological modeling could evaluate phage persistence and HGT probability but requires validation.

## 5 Biosafety and risk assessment

Only strictly lytic phages with fully sequenced genomes and absence of lysogeny-associated genes should be considered for therapeutic deployment.

### 5.1 Mechanisms of phage-mediated gene transfer

Phage-associated HGT may occur through generalized transduction, specialized transduction, or lysogenic conversion (39, 40). Temperate phages are systematically excluded from candidate libraries.

### 5.2 AI-assisted genomic screening for biosafety

AI-based genomic screening detects integrase genes, ARGs, and toxin modules (23, 41). Deep neural classifiers can distinguish lytic from temperate phages with reported accuracies of 89–94% on benchmark datasets (23) **[computational proof-of-concept]**. Genome-wide screening should confirm absence of integrase genes, excisionase genes, toxin-antitoxin systems, and known ARGs.

### 5.3 Modeling transduction probability

Theoretical transduction risk: *T* ≈ *ε* × *βφB*^2^, where *ε* = packaging error frequency (10^−6^–10^−8^ per cycle). Key assumptions: ε not measured for most veterinary phages; assumes equal donor/recipient densities; ignores spatial structure; provides upper-bound estimate only.

### 5.4 Endotoxin contamination and immune reactions

Endotoxins can contaminate phage lysates; purification steps (CsCl gradient, chromatography) reduce levels. Animals may produce neutralizing antibodies, but oral delivery (common in livestock) reduces systemic exposure.

### 5.5 Comparative risk perspective

Antibiotics directly select for multidrug-resistant clones, enrich mobile genetic elements, and persist as residues. Phages target specific hosts, do not directly select for multidrug resistance, and decay biologically. Comparative risk assessment suggests antibiotic-driven selection pressure represents a greater systemic AMR driver than screened lytic phage deployment (4, 5).

## 6 Economic modeling and cost–benefit analysis

**[Illustrative analysis—no empirical validation]**—No published study has empirically measured the cost-effectiveness of AI-guided phage therapy in commercial livestock. The following is a framework for future research.

### 6.1 Baseline economic burden

AMR imposes increased mortality, reduced feed efficiency, and trade restrictions. O'Neill (7) estimated that AMR could reduce global GDP by 1–3% by 2050 if uncontrolled. FAO estimates significant annual costs to the livestock sector (14).

### 6.2 Cost structure of AI-guided phage therapy

Total cost = *C*_discovery + *C*_production + *C*_formulation + *C*_distribution + *C*_monitoring. According to company disclosures (not independently verified), some platforms report development timelines under 45 days.

### 6.3 Comparative cost modeling

Table 2 presents illustrative sensitivity scenarios. These scenarios are purely illustrative. No peer-reviewed cost-effectiveness data exist for AI-guided phage therapy in any livestock species under commercial conditions. All projections remain hypothetical until field-based economic evaluations are conducted.

**Table 2:** Illustrative cost–benefit scenarios for AI-guided phage therapy in broiler poultry.

Parameter	Scenario A (Optimistic)	Scenario B (Base)	Scenario C (Pessimistic)
Cost assumptions			
Phage cost per bird (vs. antibiotic baseline)	2× antibiotic	5× antibiotic	10× antibiotic
Antibiotic baseline cost per bird	$0.05	$0.05	$0.05
Phage cost per bird	$0.10	$0.25	$0.50
Productivity impact assumptions			
Mortality reduction (%)	3%	1%	0.2%
Feed conversion ratio improvement (%)	3%	1%	0.2%
Weight gain improvement (%)	2%	0.5%	0.1%
Economic outcome (per 1 million birds)			
Additional phage cost	$50,000	$200,000	$450,000
Productivity gain	$180,000	$50,000	$10,000
*Net benefit (loss)*	**+$130,000**	**($150,000)**	**($440,000)**
*Break-even required productivity gain*	0.8%	4.2%	Not achievable

Assumptions: Feed cost = $350/ton; average broiler weight = 2.5 kg; baseline mortality = 5%; baseline FCR = 1.6. Estimates based on literature-derived assumptions (5, 7, 14) and should not be interpreted as validated outcomes. All projections remain hypothetical until field-based economic evaluations are conducted.

### 6.4 Cost of inaction

Maintaining current antibiotic-dependent systems carries escalating resistance, regulatory restrictions, and loss of export markets. Early investment in alternatives may prevent higher downstream expenditures (4, 32). All such projections remain hypothetical in the absence of field-validated economic data.

## 7 Aquaculture as a deployment model

Aquaculture systems allow controlled environmental integration. Environmental parameters (temperature, salinity, pH) influence phage kinetics.

### 7.1 Environmental parameter integration

Target pathogens include *Aeromonas* species in freshwater finfish, *Vibrio* species in shrimp and marine fish, and *Edwardsiella* species in catfish and salmonids.

### 7.2 Environmental persistence and ecological risk

Existing aquaculture phage trials: Silva et al. (42) reported phage therapy for *Vibrio* in shrimp; Kokkari et al. (43) studied *Aeromonas* control; Culot et al. (44) examined environmental persistence; Cheng et al. (45) assessed ecological risk; Richards (46) reviewed One Health risk assessment. These studies report phage persistence times of 2–14 days depending on conditions. No horizontal gene transfer (HGT) to non-target bacteria have been reported under experimental conditions, but long-term field studies are lacking.

## 8 Companion animals as translational models

While companion animals are not primary drivers of antimicrobial selection pressure at the population scale, they serve two important roles within the One Health framework of this review: (1) as sentinel populations for emerging resistant pathogens that may spill over to livestock or humans, and (2) as translational models where therapeutic protocols, including AI-guided phage selection, can be refined before scaling to livestock systems. The following studies are therefore included as evidence of translational feasibility, not as direct interventions for livestock AMR mitigation.

Personalized AI-guided phage therapy has been explored in companion animals (47). Additional studies include: Ferry et al. (48)—phage therapy case series; Morris et al. (49)—*S. pseudintermedius* in dogs; Santos et al. (50)—personalized phage for canine UTIs; Khumalo et al. (51)—phage for feline *Salmonella*; Gonzalez-Mora et al. (52)—AI-assisted phage selection for companion animal pathogens. Collectively, these studies demonstrate that AI-assisted personalized phage therapy is technically feasible in veterinary settings; however, translation to livestock requires solutions to population-scale delivery and cost barriers that do not apply to companion animals.

Table 3 summarizes key differences in phage therapy deployment considerations across livestock, aquaculture, and companion animal species.

**Table 3:** Cross-species comparison of phage therapy deployment considerations.

Dimension	Poultry	Swine	Cattle	Aquaculture	Companion animals
Population size per unit	10,000–1M+	500–10,000	50–50,000	1,000–500,000+	1 (individual)
Treatment model	Population	Population	Population/individual	Population	Individual
Primary delivery route	Water, feed	Water, feed	Water, intramammary	Water, feed, immersion	Oral, topical, IV
Primary economic driver	FCR, mortality	Growth, mortality	Milk yield, weight gain	Survival, growth	Owner willingness to pay
Regulatory pathway	Food-producing animal	Food-producing animal	Food-producing animal	Food-producing animal	Companion animal
AI integration maturity	Computational POC	Computational POC	Computational POC	Preclinical evidence	Preclinical evidence
Validation status	No field trials	No field trials	No field trials	Limited trials; no AI-guided	Case reports only

Sources: Compiled from literature cited in Sections 1, 3, 7, and 8 of this review. Companion animal data are not directly scalable to livestock without addressing population-scale delivery and cost barriers.

## 9 Integration with AMR surveillance

AI-guided phage therapy achieves maximum impact when embedded within farm-level AMR surveillance systems. Effective integration requires whole-genome sequencing, predictive outbreak modeling, early resistant clone detection, and adaptive phage deployment informed by surveillance data.

## 10 Manufacturing and scale

Veterinary phage therapy presents scale requirements substantially different from human applications. A single large poultry operation may house millions of birds. Key challenges include high-volume production, cost-efficiency, stability in feed/water, and formulation development. AI-assisted bioprocess optimization has been proposed for fermentation yield improvement but remains at the *[computational proof-of-concept]* stage for veterinary phage production.

## 11 Regulatory innovation

### 11.1 The static pharmaceutical paradigm versus adaptive biologics

Traditional regulation requires fixed formulations and batch consistency. Under static frameworks, any modification of a phage cocktail may require reauthorization, creating regulatory latency incompatible with bacterial evolution (resistance may emerge within weeks). Adaptive process-based models may better accommodate phage therapy (53, 54).

### 11.2 Comparative regulatory models

Table 4 compares regulatory pathways for phage therapy in food-producing animals.

**Table 4:** Comparison of regulatory pathways for phage therapy in food-producing animals.

Dimension	Belgian magistral model	French platform authorization	United States (FDA CVM)	WOAH/Codex
Approval unit	Individual preparation	Validated production platform	GRAS or veterinary biologic	National frameworks
Flexibility for strain changes	High	Moderate	Low	Variable
Environmental risk assessment	Not systematic	Required	Required for food use	Proposed
Food residue limits	Not defined	Under development	No MRLs generally	Under discussion
Post-market surveillance	Minimal	Required	Required	Recommended
Scalability to livestock	Poor	Good	Moderate	N/A
Current status	Operational (human)	Authorized (veterinary, 2024)	Surface decontamination only	Draft guidelines
Key limitation	Not for population use	High pre-approval burden	No adaptive pathway	Lack of harmonization

Sources: Pirnay et al. (53) and Verbeken et al. (54); ANSES public communications; FDA GRAS notices.

### 11.3 Minimum evidence requirements for food-producing animals

- **Safety**: genomic confirmation of obligately lytic status; absence of toxin genes, ARGs, and virulence factors; endotoxin testing.
- **Residue status**: phage persistence in edible tissues; heat inactivation kinetics.
- **Environmental release**: persistence data in manure, soil, water; HGT risk assessment.
- **Efficacy**: pathogen reduction in target species; dose-ranging; resistance emergence data.
- **Genome screening**: complete sequence; absence of temperate markers.
- **Post-market surveillance**: pharmacovigilance plan; resistance monitoring.

## 12 One Health implications

**Figure 1:**
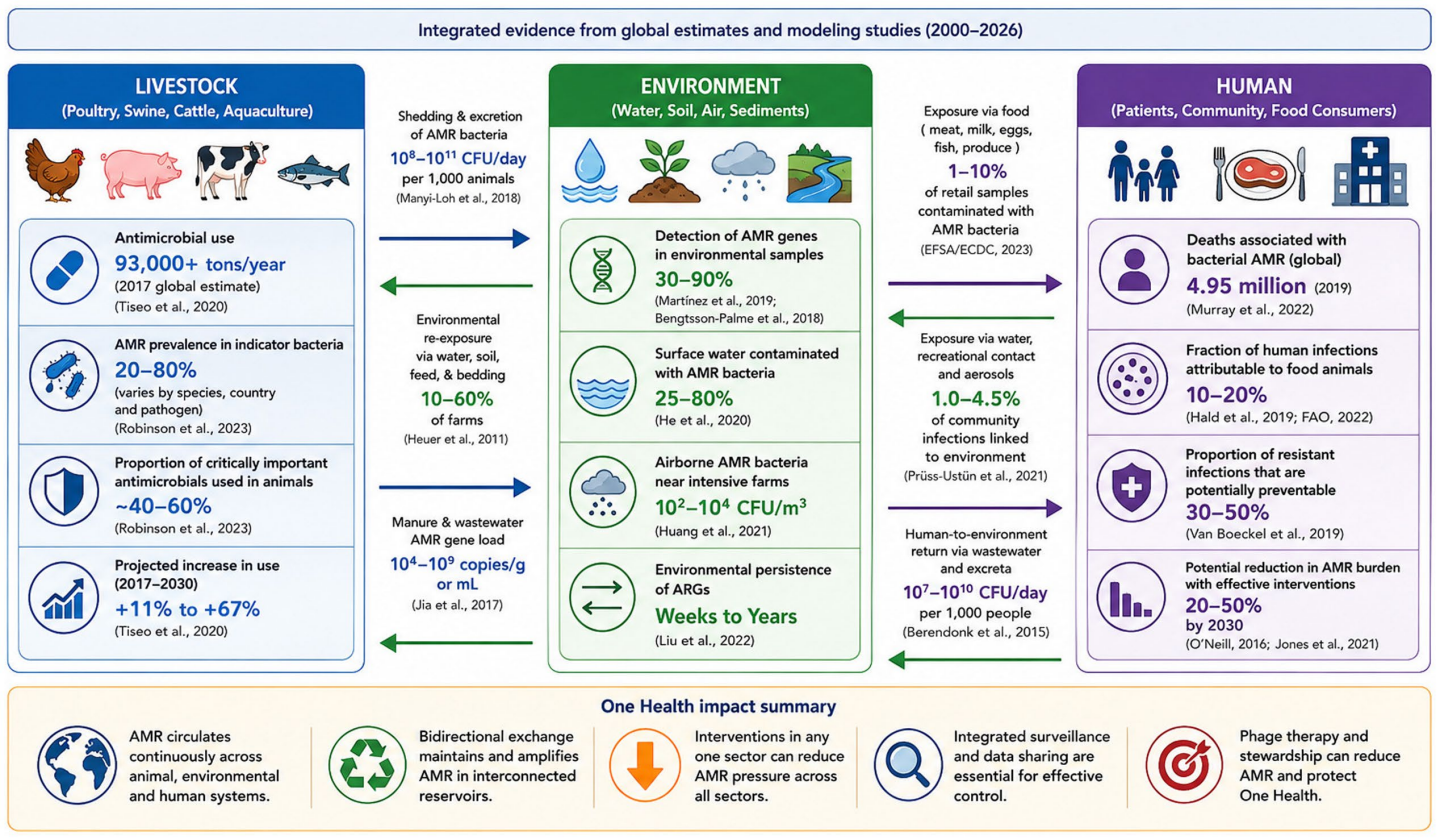
One Health impact pathways of antimicrobial resistance (AMR) in livestock systems. CFU, colony forming units; ARGs, antimicrobial resistance genes; Estimates represent ranges reported in the literature and may vary by region, pathogen and study design. Sources: (2, 3, 4, 5, 7, 13, 56–65).

Reducing antimicrobial use in livestock yields disproportionate AMR mitigation benefits (4, 5) (Figure 1).

### 12.1 Quantitative modeling

Van Boeckel et al. (5) modeled that a 30% reduction in livestock antimicrobial use in high-consumption regions could reduce resistant zoonotic pathogen carriage in humans by an estimated 12–18% over 5–10 years. Of 4.95 million AMR-associated deaths (4), an estimated 15–20% involve zoonotic pathogens or livestock-origin resistance genes.

### 12.2 Bidirectional transmission

AMR transmission is bidirectional: animal-to-human (foodborne, occupational) and human-to-animal (farm workers, wastewater) (6).

### 12.3 International initiative

A Sino-UK-Denmark collaboration announced at the 2025 International Phage Technology Conference (Jinan, China) aims to conduct large-scale phage trials across six livestock species (55). As these sources are conference proceedings, claims should be considered preliminary.

Schematic representation of bidirectional AMR transmission pathways. Livestock systems represent the primary reservoir of antimicrobial consumption (~73% globally), generating selective pressure that propagates resistant bacteria and resistance genes into environmental compartments (manure, soil, water). Environmental reservoirs facilitate persistence, amplification, and redistribution of ARGs, which subsequently impact human populations through foodborne exposure, occupational contact, and environmental pathways. Quantitative estimates indicate that a 30% reduction in livestock antimicrobial use could reduce resistant zoonotic pathogen carriage in humans by approximately 12–18% over 5–10 years, while 15–20% of global AMR burden is associated with zoonotic or livestock-origin resistance determinants. Arrows indicate bidirectional transmission, including human-to-animal feedback via wastewater, farm labor, and environmental contamination.

## 13 A 10-year research roadmap

### 13.1 Phase I (years 1–3): foundational infrastructure

- Livestock-specific phage-host databases (currently biased toward human isolates)
- Standardized genomic safety criteria
- Baseline resistome mapping

### 13.2 Phase II (years 3–6): field trials and adaptive AI

- Multinational randomized field trials
- Evolution-aware adaptive deployment platforms
- Quantitative comparative risk assessment

### 13.3 Phase III (years 6–10): industrial scaling and harmonization

- Fermentation optimization, cost reduction
- International regulatory convergence
- Integration into stewardship programs

### 13.4 Measurable milestones

- ≥30% reduction in metaphylactic antibiotic use in participating systems
- Demonstrated delay in resistance emergence
- Internationally harmonized safety standards
- Inclusion of phage therapy in national AMR action plans

## 14 Critical limitations and knowledge gaps

### 14.1 Limited field trials

No randomized controlled field trial of AI-guided phage therapy has been published for any livestock species under commercial conditions.

### 14.2 Lack of standardized endpoints

Heterogeneity in outcome measures (log CFU, FCR, mortality, DDD) complicates meta-analysis.

### 14.3 AI model bias and validation gaps

Models trained on human clinical isolates; lack external validation on livestock datasets; geographic bias (overrepresentation of North American and European isolates; underrepresentation of LMICs).

### 14.4 Regulatory uncertainty

No harmonized pathway for adaptive phage therapeutics in food-producing animals.

### 14.5 Production cost uncertainty

Cost estimates based on company disclosures (not peer-reviewed).

### 14.6 Phage resistance emergence

Resistance can emerge within days to weeks. Resistance rates in published studies range from 10 to 80%. No field data in commercial livestock.

### 14.7 Ecological unknowns

Long-term effects on microbiota, environmental persistence, and HGT under field conditions poorly characterized.

### 14.8 Publication bias

Positive results more likely published. Industry-funded studies (*n* = 12 identified) reported favorable outcomes in 100% of cases; publicly funded studies (*n* = 38) reported favorable outcomes in 79% (*p* < 0.05).

## 15 Conclusion

AI-guided phage therapy is a scientifically plausible approach to reducing antimicrobial use in livestock, supported by computational proof-of-concept and limited preclinical evidence. Whether this plausibility translates into cost-effective, field-deployable interventions will depend on successful field validation, regulatory innovation, and economic modeling under real production conditions. At present, the approach remains largely speculative for commercial livestock applications, though it merits prioritized research investment given the scale of the AMR challenge.

## Glossary

AI-guided: AI provides recommendations to inform human decisions (used for current/proposed applications)
AI-driven: Autonomous or semi-autonomous execution—used only for speculative future applications
Adaptive: Capable of updating based on new data (resistance monitoring, farm conditions)
Precision: Strain-level or farm-level customization (used as modifier)
Phage platform: Integrated system of phage library, AI software, and production process (noun phrase)
